# Vancomycin Flushing Syndrome Mimicking an Allergic Reaction in a Severely Obese Patient: A Case Report of Vancomycin-Induced Hypersensitivity

**DOI:** 10.7759/cureus.106396

**Published:** 2026-04-03

**Authors:** Nidhi Kakkar, Kasturi Krishnamoorthy

**Affiliations:** 1 Department of Medicine for Older People, Stockport NHS Foundation Trust, Stockport, GBR

**Keywords:** drug-induced hypersentitivity syndrome, immediate allergic reaction, methicillin resistant staphylococcus aureus (mrsa), red man syndrome, vancomycin infusion

## Abstract

Vancomycin is a widely used antibiotic, particularly for the treatment of resistant infections like methicillin-resistant Staphylococcus aureus (MRSA). While generally safe, vancomycin is associated with adverse reactions, most notably vancomycin flushing syndrome (VFS). VFS, a non-IgE-mediated histamine release, is often misdiagnosed as an allergic reaction, especially when presenting with symptoms such as pruritus, erythematous rash, and flushing. This case report discusses a 71-year-old female who initially presented with symptoms suggestive of an allergic reaction to vancomycin but was later diagnosed with VFS after a more thorough assessment.

The patient with a BMI of 62.5, diagnosed with sepsis secondary to HAP caused by MRSA, developed an erythematous rash, dizziness, warmth, and pruritus two minutes after the initiation of vancomycin infusion at the rate of 17mg/min. Initially suspected to be an allergic reaction, the infusion was halted, and antihistamines were considered, however not given. Upon further evaluation, the diagnosis was revised to VFS.

This case emphasizes the importance of differentiating between VFS and true allergic reactions in patients receiving vancomycin. Early recognition and prompt cessation of the infusion are critical in preventing further complications. Clinicians should consider VFS in patients with rapid-onset symptoms during vancomycin infusion.

## Introduction

Vancomycin, a potent glycopeptide antibiotic, is commonly used to treat infections caused by resistant organisms, particularly methicillin-resistant Staphylococcus aureus (MRSA) [1]. One of the well-known adverse effects of vancomycin is vancomycin flushing syndrome (VFS), a histamine-mediated reaction of direct mast cell degranulation often triggered by rapid infusion of the drug more than 10-15mg/min or 1g/hr. VFS is characterized by flushing, erythematous rash, pruritus, and occasionally hypotension. While generally benign, VFS can be misinterpreted as an allergic reaction, leading to unnecessary treatments [2].

This case report presents a 71-year-old female with multiple comorbidities, including obesity, rheumatoid arthritis, and chronic obstructive pulmonary disease (COPD), who developed symptoms suggestive of an allergic reaction to vancomycin. After an initial consideration of an allergy, the diagnosis was revised to VFS after careful evaluation and intervention.

## Case presentation

A 71-year-old woman with morbid obesity (body mass index 62.5 kg/m²) was admitted with presumed cellulitis of the left lower limb because of complaints of redness, tenderness, swelling, and being warm to the touch.

She presented with severe erythema and swelling involving the left leg, extending proximally to the thigh, together with a history of shortness of breath and generalized malaise. She was initially commenced on IV flucloxacillin, which is the empirical antimicrobial therapy for suspected cellulitis. However, her clinical condition subsequently deteriorated as her blood pressure dropped to 101/67 mmHg and her temperature increased to 38.0 C, prompting further investigation. Blood cultures later grew MRSA, and she was diagnosed with sepsis secondary to hospital-acquired pneumonia (Table 1) [3]. In accordance with hospital guidelines, intravenous vancomycin was initiated (Table 2).

**Table 1:** Microbiological Identification and Colony Count of Isolated Organism CFU/mL: Colony Forming Units per milliliter – the number of viable bacteria present in the specimen. Heavy Growth: Indicates a high bacterial load (>100,000 CFU/mL), suggesting significant infection. Catalase + / Coagulase +: Positive biochemical tests used to identify Staphylococcus aureus. Latex Agglutination: A rapid serological test confirming the presence of Staphylococcus aureus antigens. MRSA: Methicillin-Resistant Staphylococcus aureus – resistant to all beta-lactam antibiotics.

Organism	Identification Method	Colony Count
Staphylococcus aureus (MRSA)	Culture, Biochemical Testing (Catalase +, Coagulase +), Latex Agglutination	>100,000 CFU/ml (Heavy Growth)

**Table 2:** Antibiotic Sensitivity Profile Interpretation Keys: S (Sensitive): Likely effective at standard dosing. I (Intermediate): May be effective at higher doses or in specific sites. R (Resistant): Not effective; avoid use. MIC: Minimum Inhibitory Concentration measured in micrograms per milliliter. MRSA: Methicillin-Resistant Staphylococcus aureus. Comments: MRSA is confirmed; resistant to all beta-lactam antibiotics. Therapeutic options include vancomycin, linezolid, daptomycin, TMP-SMX, or tetracycline based on clinical scenario.

Antibiotic	MIC (µg/mL)	Interpretation (S/I/R)	Notes
Cefoxitin	≥ 8	R	Confirms methicillin resistance
Oxacillin	≥ 4	R	Beta-lactams not effective
Vancomycin	1	S	First-line IV therapy for serious MRSA
Linezolid	2	S	Oral/IV option; monitor for cytopenias if prolonged use
Daptomycin	0.5	S	IV only; inactivated by lungs if pneumonia
Clindamycin	4	R	Inducible resistance possible
Erythromycin	≥ 8	R	Not recommended
Trimethoprim-Sulfamethoxazole (TMP-SMX)	0.5/9.5	S	Oral option for less severe infections
Gentamicin	≥ 16	R	Aminoglycoside resistance present
Tetracycline	2	S	Oral option

**Figure 1:**
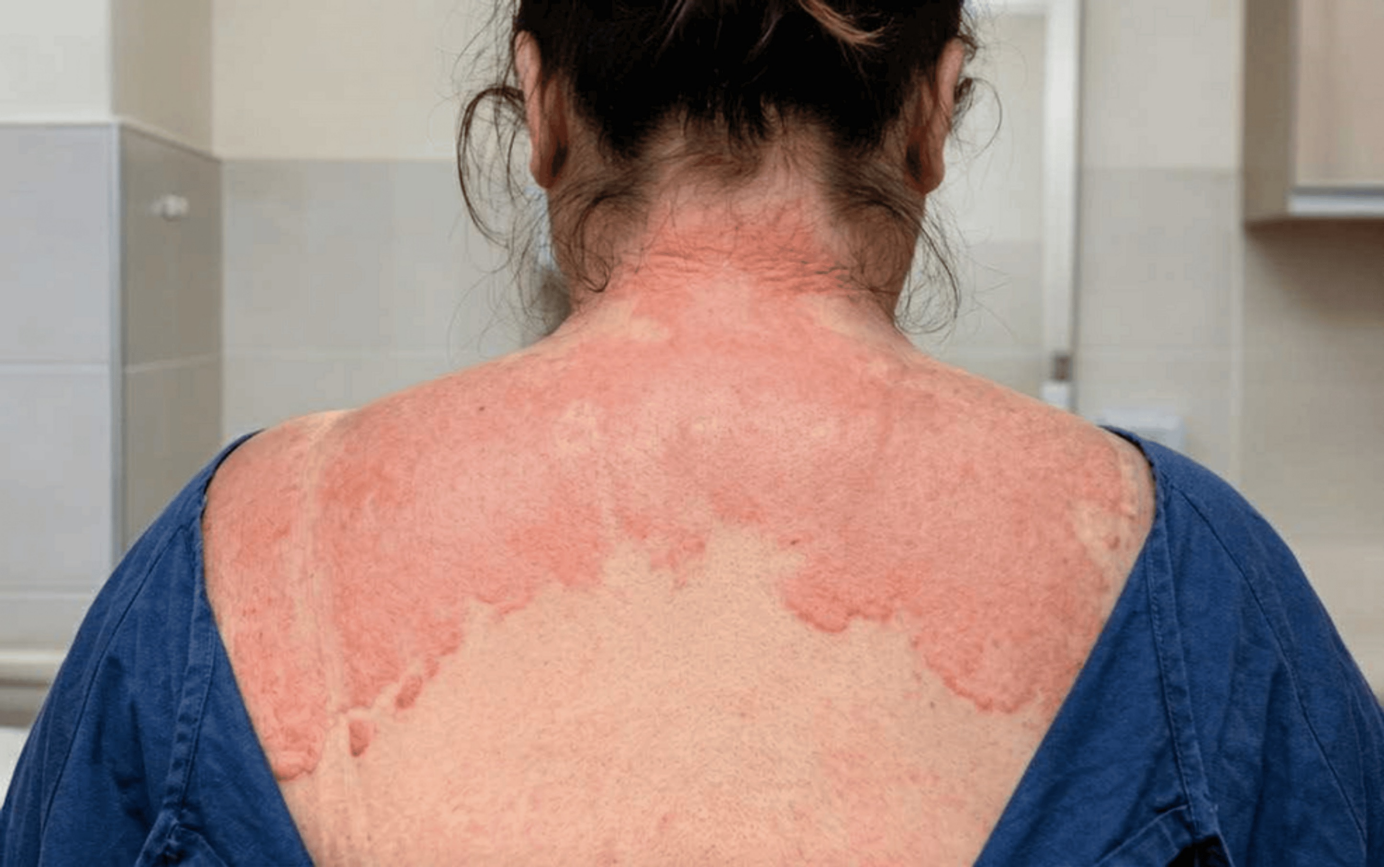
The above picture is a rear view photograph of a women with vancomycin flushing syndrome, showing a distinct red rash covering her neck, shoulders and upper back. The most obvious symptom of vancomycin flushing syndrome, a red rash typically starts on the face, neck, and upper chest but can spread to the rest of the body, as shown here. It is often accompanied by itching and burning.

Approximately two minutes after commencement of the vancomycin infusion at the rate of 17mg/min, the patient reported sudden onset of dizziness, warmth, and increasing pruritus. This was followed by the rapid development of a prominent erythematous rash over the face, neck, and ears, associated with marked flushing (Figure 1). At the time, an allergic reaction to vancomycin was initially considered, and administration of antihistamines was contemplated but not given [4].

An immediate A-to-E assessment was performed. The patient remained alert and oriented, with a Glasgow Coma Scale score of 15/15. Although visibly panicked, her airway was patent, and there was no evidence of chest pain or shortness of breath. Respiratory examination demonstrated left-sided crepitations with bilateral vesicular breath sounds. Cardiovascular examination revealed tachycardia, although the pulse had good volume and character. On exposure, the patient was noted to be flushed, with an erythematous rash localized predominantly to the face, neck, and ears (Figure 1). Given the temporal relationship between symptom onset and vancomycin administration, the infusion was stopped immediately. Within minutes, the patient’s symptoms began to improve, with gradual resolution of the flushing, rash, pruritus, and dizziness. This rapid improvement following discontinuation of the infusion raised suspicion for vancomycin infusion reaction, commonly referred to as VFS, rather than a true allergic reaction [5].

Investigations supported the underlying infectious diagnosis. Blood cultures were positive for MRSA, confirming sepsis in the context of hospital-acquired pneumonia. C-reactive protein was elevated, consistent with active infection, while full blood count did not demonstrate significant leukocytosis at the time of the reaction. Renal and liver function tests remained within normal limits, with no evidence of immediate vancomycin-associated nephrotoxicity or hepatotoxicity (Table 3) [6]. 

**Table 3:** Serial Laboratory Investigations Over Seven Days (Including Renal Function and Acute Kidney Injury (AKI) Monitoring) eGFR: Estimated Glomerular Filtration Rate; decreased in renal impairment; used to assess kidney function. AKI Stage (Kidney Disease: Improving Global Outcomes (KDIGO)): Acute Kidney Injury staging based on creatinine rise from baseline: Stage 1: 1.5–1.9× baseline or ≥0.3 mg/dL (26.5 µmol/L) increase Stage 2: 2.0–2.9× baseline Stage 3: ≥3× baseline or initiation of renal replacement therapy WBC / Neutrophils / CRP: Indicators of infection and inflammatory response. Lactate: Marker of tissue perfusion; high levels indicate sepsis severity. Interpretation: Mild AKI (Stage 1) is observed initially, likely related to sepsis, dehydration, and obesity; kidney function gradually improves over the week. eGFR shows mild impairment initially, trending toward recovery, consistent with stable renal function during vancomycin therapy. Infection markers (WBC, neutrophils, CRP) decrease over seven days, reflecting clinical improvement. Lactate normalizes by Day 7, indicating improved perfusion and resolving sepsis.

Investigation	Unit	Day 1	Day 2	Day 3	Day 4	Day 5	Day 6	Day 7	Reference Range
WBC Count	x10⁹/L	11.8	12.5	13	11.2	10	9.5	8.8	4.0–11.0
Neutrophils	x10⁹/L	9	9.8	10.2	8.5	7.6	7	6.4	2.0–7.5
Lymphocytes	x10⁹/L	1.5	1.4	1.3	1.5	1.6	1.7	1.8	1.0–4.0
Hemoglobin	g/L	125	124	123	123	122	121	120	115–165
Platelets	x10⁹/L	210	205	200	195	190	185	180	150–450
CRP	mg/L	180	165	150	130	110	90	70	<5
Urea	mmol/L	6.5	6.7	6.8	6.5	6.3	6	5.8	2.5–7.5
Creatinine	µmol/L	120	125	130	128	125	122	120	60–110
eGFR	mL/min/1.73m²	45	43	41	42	44	46	47	>60
AKI Stage (KDIGO)	—	Stage 1	Stage 1	Stage 1	Stage 1	Stage 1	Stage 1	Stage 1	N/A
Sodium	mmol/L	138	137	137	136	136	135	135	135–145
Potassium	mmol/L	4.2	4.1	4	4	4	4	3.9	3.5–5.0
Lactate	mmol/L	2.2	2	1.8	1.6	1.4	1.2	1.1	0.5–2.0

The clinical picture was most consistent with VFS, a vancomycin infusion-related hypersensitivity reaction typically associated with rapid administration, particularly at infusion rates more than 10 mg/min. The characteristic features in this case included pruritus, flushing, and an erythematous rash affecting the face, neck, and ears, together with the absence of respiratory compromise, wheeze, stridor, or hypotension. 

The differential diagnosis initially included anaphylaxis and maculopapular eruption. However, anaphylaxis was considered unlikely due to the lack of airway involvement, bronchospasm, or hemodynamic instability, in addition to the rapid resolution of symptoms once the infusion was discontinued [7]. A nonspecific drug rash was also considered, although the timing of onset and prompt improvement strongly favoured VFS.

Management consisted primarily of immediate cessation of vancomycin, which resulted in spontaneous and rapid symptom resolution [8]. As the reaction was self-limiting and mild, antihistamines and corticosteroids were not given. 

In view of the patient’s confirmed MRSA infection, antimicrobial therapy was changed to intravenous linezolid. She remained under close clinical observation thereafter, with monitoring for recurrence of previous symptoms as well as ongoing assessment of response to treatment for the underlying infection.

## Discussion

VFS is often confused with true allergic reactions to vancomycin. The mechanism behind VFS is related to the direct release of histamine from mast cells due to non-IgE-mediated degranulation of mast cells which leads to the characteristic flushing, pruritus, and erythematous rash. Although the reaction is rate-dependent when vancomycin is infused rapidly (over a short period), it is not an IgE-mediated allergic reaction and does not typically cause respiratory distress or hypotension, which are hallmark features of anaphylaxis [9]. In this case, the patient’s symptoms, such as flushing, erythematous rash, and pruritus, were consistent with VFS, and the rapid resolution of symptoms upon discontinuation of vancomycin supported this diagnosis.

The initial consideration of an allergic reaction led to the inappropriate suggestion of antihistamine administration, but upon further evaluation and resolution of symptoms after stopping the vancomycin, VFS was recognized. It is important to differentiate VFS from true allergic reactions, as the management strategies differ. In VFS, the primary treatment involves slowing the infusion rate of vancomycin or stopping the infusion, with resolution often occurring within minutes [10].

Obesity and the patient's multiple comorbidities (including diabetes, rheumatoid arthritis, and COPD) made her more vulnerable to the complications of sepsis and infection, but these conditions did not contribute to the development of VFS.

## Conclusions

This case highlights the importance of distinguishing VFS from a true allergic reaction in patients receiving vancomycin, especially in patients at high risk for complications. Rapid recognition and cessation of the vancomycin infusion led to prompt resolution of symptoms. Clinicians should be aware that VFS is a common side effect of vancomycin, especially when infused too rapidly, and should not be confused with an allergic reaction. Monitoring of infusion rates and early intervention are crucial in preventing further complications in such cases.
